# Identification of mitophagy-related key genes and their correlation with immune cell infiltration in acute myocardial infarction via bioinformatics analysis

**DOI:** 10.3389/fcvm.2024.1501608

**Published:** 2025-01-13

**Authors:** Zulong Sheng, Rui Zhang, Zhenjun Ji, Zhuyuan Liu, Yaqing Zhou

**Affiliations:** Department of Cardiology, Zhongda Hospital, Medical School of Southeast University, Nanjing, China

**Keywords:** mitophagy, acute myocardial infarction, bioinformatics analysis, biomarkers, diagnostic risk model

## Abstract

**Background:**

Acute myocardial infarction (AMI), a subset of acute coronary syndrome, remains the major cause of mortality worldwide. Mitochondrial dysfunction is critically involved in AMI progression, and mitophagy plays a vital role in eliminating damaged mitochondria. This study aimed to explore mitophagy-related biomarkers and their potential molecular basis in AMI.

**Methods:**

AMI datasets (GSE24519 and GSE34198) from the Gene Expression Omnibus database were combined and the batch effects were removed. Differentially expressed genes (DEGs) in AMI were selected, intersected with mitophagy-related genes for mitophagy-related DEGs (MRDEGs), and then subjected to enrichment analyses. Next, the MRDEGs were screened using machine learning methods (logistic regression analysis, RandomForest, least absolute shrinkage and selection operator) to construct a diagnostic risk model and select the key genes in AMI. The diagnostic efficacy of the model was evaluated using a nomogram. Moreover, the infiltration patterns of different immune cells in two risk groups were compared. We also explored the interactions between the key genes themselves or with miRNAs/transcription factors (TFs) and drug compounds and visualized the protein structure of the key genes. Finally, we explored and validated the expression of key genes in plasma samples of patients with an AMI and healthy individuals.

**Results:**

We screened 28 MRDEGs in AMI. Based on machine learning methods, 12 key genes were screened for the diagnostic risk model, including *AGPS, CA2, CAT, LTA4H, MYO9B, PRDX6, PYGB, SIRT3, TFEB, TOM1, UBA52*, and *UBB*. The nomogram further revealed the accuracy of the model for AMI diagnosis. Moreover, we found a lower abundance of immune cells such as gamma delta T and natural killer cells in the high-risk group, and the expression of key genes showed a significant correlation with immune infiltration levels in both groups. Finally, 64 miRNA–mRNA pairs, 75 TF–mRNA pairs, 119 RNA-binding protein–mRNA pairs, and 32 drug–mRNA pairs were obtained in the interaction networks.

**Conclusions:**

In total, 12 key MRDEGs were identified and a risk model was constructed for AMI diagnosis. The findings of this study might provide novel biomarkers for improving the detection of AMI.

## 1 Introduction

Acute myocardial infarction (AMI) is a major cause of hospitalization and death across the world, with an increasing incidence annually (1). According to the Report on Cardiovascular Health and Diseases in China, there were over one million hospitalized patients with an AMI in 2022, and the in-hospital mortality was 4.3% (2). As a serious coronary heart syndrome, AMI can cause life-threatening symptoms such as sudden cardiac death and cardiogenic shock (3, 4). In the past decades, myocardial reperfusion, especially percutaneous coronary intervention, and new thrombolytic drugs have significantly improved the survival of patients with an AMI (5). However, these therapies can induce ischemia–reperfusion injury and other pathologies, and the incidence of heart failure has still increased annually since their introduction (6). Effective and reliable methods for the early detection of AMI are still not available (7, 8). Exploration of effective biomarkers for the accurate prediction of AMI is the key to the prevention and management of AMI.

Mitochondria constitute approximately 30% of total cardiomyocyte volume. Apart from producing adenosine triphosphate (ATP) via oxidative phosphorylation, mitochondria are also responsible for cellular reactive oxygen species (ROS) production (9). Damage to mitochondria can cause excessive production of ROS and lead to oxidative stress, cell injury, and cell death, as well as inflammatory responses and immune cell activation, contributing to the pathological processes of cardiovascular diseases (10, 11). Mitochondrial dysfunction is a crucial driver of heart injury in ischemic heart disease, thus exploring strategies to maintain mitochondrial homeostasis is of vital significance for intervention in cardiovascular diseases. Mitophagy is a selective and specialized autophagic pathway that regulates mitochondrial quality and quantity by removing or degrading dysfunctional or redundant mitochondria and is essential for maintaining mitochondrial and cellular homeostasis (12–14).

Studies have revealed the pivotal role of mitophagy in heart diseases, including myocardial ischemia, by removing malfunctioning mitochondria, thus alleviating oxidative stress, improving cell survival, and mitigating heart injury (15–17). Additionally, AMI is associated with immune cell infiltration in the acute and reparative phases, while autophagy has been found to regulate the immune system by mediating mitochondrial homeostasis (18). In AMI, mitophagy is activated via multiple signaling such as the Parkin- and receptor-mediated pathways and the Ulk1/Rab9/Rip1/Drp1 pathway (19). Mitophagy-related genes (MRGs) have been revealed to regulate this physiological process and their dysregulation affects AMI progression, suggesting their roles as potential targets for AMI prevention and treatment (19–21). However, mitophagy-related biomarkers in AMI have not been fully elucidated. The expression profile and relationship of MRGs in AMI remain largely unknown.

Therefore, this study intended to identify mitophagy-related key genes and evaluate their value for AMI diagnosis. We analyzed the differentially expressed genes (DEGs) in AMI and intersected these DEGs with mitophagy-related genes to find mitophagy-related DEGs (MRDEGs). A risk model was constructed using the MRDEGs via machine learning methods, and a nomogram was used to assess the diagnostic accuracy of the model. Moreover, the correlations between the key MRDEGs with immune infiltration and relevant networks were explored. Additionally, a transcription factor (TF)-miRNA network and a protein-drug network for the key genes were constructed, which might provide novel insight into the molecular basis of MRDEGs involved in AMI.

## 2 Materials and methods

### 2.1 Data acquisition and pre-processing

**Figure 1:**
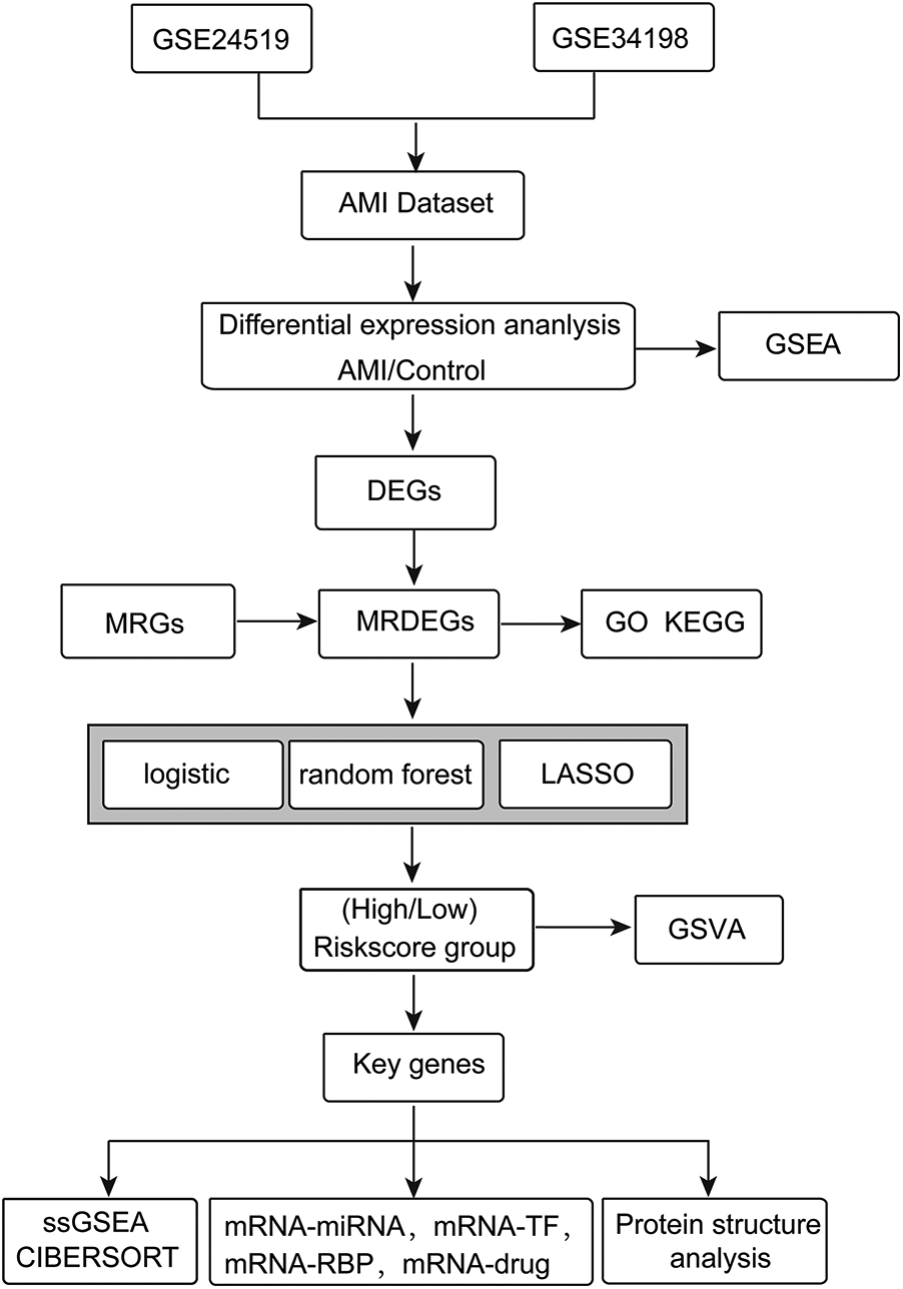
The workflow of this study.

Human blood gene expression profiling datasets (GSE24519 and GSE34198) were downloaded from the Gene Expression Omnibus (GEO) database (22) using the “GEOquery” R package (23). The GSE24519 bioarray dataset included 34 AMI blood samples and 4 normal blood samples from the GPL2895 platform. The GSE34198 dataset included 49 AMI blood samples and 48 normal samples from the GPL6102 platform. The information from the two datasets is shown in Supplementary Table 1. The workflow of our study is shown in Figure 1.

### 2.2 MRDEGs in AMI

The GeneCards database (https://www.genecards.org/) was used to screen MRGs using “Mitophagy” as the search term, and the screening criteria of “Protein Coding” and “Relevance score” >1. A total of 1,592 MRGs were obtained. In addition, we also obtained nine MRGs from previous literature. Finally, 1,593 MRGs were obtained after removing duplicates.

Before analyzing the DEGs, two GEO datasets (GSE24519 and GSE34198) were combined and debatched using the “sva” R package (24). To assess the biological variability between samples and verify the effects of debatching, principal component analysis (PCA) was performed. PCA is a commonly used method for dimensionality reduction, extracting the principle components from the high-dimensional data and retaining the trends and patterns of the original data (25). Next, the DEGs between the AMI samples and control samples were obtained with the criteria of |logFC|>0 and *p* < 0.05 using the “limma” R package (26). The collected DEGs were intersected with the MRGs to obtain the MRDEGs in AMI. The results of the differential expression analysis were visualized in a volcano plot and a heatmap using the “ggplot2” (27) and “pheatmap” R packages. The group comparison chart showing the expression pattern of MRDEGs in the control and AMI groups was produced using the “ggplot2” R package. Genes with statistically significant expression in AMI were selected for subsequent analyses. Spearman’s correlation analysis determined the expression correlation between MRDEGs. A chromosome localization map of MRDEGs was visualized using the “RCircos” R package (28).

### 2.3 Functional enrichment analyses of MRDEGs

Gene Ontology (GO) and Kyoto Encyclopedia of Genes and Genomes (KEGG) pathway enrichment analyses were conducted for the MRDEGs using the “clusterProfiler” R package (29) with a false discovery rate (FDR) value (qvalue) <0.05 and *p*.adj <0.05 using Benjamini–Hochberg (BH) as the *p*-value correction method.

### 2.4 Gene set enrichment analysis

To analyze the differences in biological processes (BPs) between AMI samples and normal samples, the “c2.cgp.v2022.1.Hs.symbols.gmt [Chemical and Genetic Perturbations] (3399)” reference geneset was downloaded from the MSigDB database (http://www.gsea-msigdb.org/gsea/msigdb/) (30, 31). Based on the gene expression profiling, we conducted the enrichment analysis using the “clusterProfiler” R package with the criteria of *p*.adj <0.05 and FDR value (qvalue) <0.05, with BH as the *p*-value correction method.

### 2.5 Selection of key genes and risk model construction via machine learning

To construct a diagnostic model for AMI, a logistic regression analysis of the association between the MRDEGs and AMI was performed, and the MRDEGs were screened using the criteria of *p* < 0.05. The results were visualized using a forest plot.

RandomForest (RF) is an algorithm that integrates multiple decision trees based on ensemble learning. The “randomForest” package (32, 33) was used to construct the risk model based on the MRDEG expression in the AMI datasets, under the conditions of set.seed (150) and ntree = 2,000. MeanDecreaseGini refers to the average reduction of the Gini coefficient. The Gini coefficient represents the impurity of the node, and MeanDecreaseGini represents the average decrease in the impurity of the variable separating nodes of all trees and indicates the importance of the variables to our grouping. Ten-fold cross-validation was then performed five times and the number of variables were selected based on the cross-validation curve. The key variables were selected based on the cross-validation and the MeanDecreaseGini value.

Least Absolute Shrinkage and Selection Operator (LASSO) is a dimensionality reduction method for selecting optimized variables. Based on the LASSO regression, some coefficients were reduced to zero, so only variables with non-zero regression coefficients were selected for the construction of the risk model (34). LASSO analysis of the MRDEGs selected using random forest was performed using the “glmnet” R package (35). The LASSO risk model was constructed and the LASSO risk score (RiskScore) was calculated using the following formula:

$$\mathrm{RiskScore}=\underset{i}{\sum }\;\mathrm{Coefficient}\;(\mathrm{gen}e_{i})\times \mathrm{mRNA}\;\mathrm{Expression}\;(\mathrm{gen}e_{i})$$

AMI patients were divided into a high- or low-risk group based on the median RiskScore. MRDEGs selected using the LASSO risk model were regarded as the key genes for the following analysis.

### 2.6 Nomogram

The diagnostic performance of the key genes was evaluated using a nomogram (36). On the basis of the LASSO results, a nomogram was drawn using the “rms” R package to reveal the relationship between the expression of the key MRDEGs and AMI diagnosis. A calibration curve was produced via calibration analysis to evaluate the accuracy and the resolution of the LASSO risk model on the basis of the key MRDEGs. A decision curve analysis (DCA) diagram was drawn to evaluate the accuracy and discrimination of the LASSO risk model using the “ggDCA” R package.

To quantitatively measure the semantic similarity of the MRDEGs, the GO terms were compared using the “GOSemSim” R package (37, 38), followed by the calculation of the geometric mean of the MRDEGs at the three levels [BP, cellular component (CC), and molecular function (MF)] to obtain the final score. The MRDEGs were then sorted in descending order. The visualization of the results was conducted using the “ggplot2” R package.

### 2.7 Gene set variation analysis

Gene set variation analysis (GSVA) is a non-parametric and unsupervised method for evaluating the enrichment of a transcriptome gene set (39). The “h.all.v7.4.symbols.gmt” geneset in the MSigDB database was acquired, and GSVA was conducted for all genes in the two risk groups. The difference in the functional enrichment of genes between the two risk groups was analyzed with the criteria of *p* < 0.05. Finally, the enriched pathways via GSVA were displayed.

### 2.8 Analysis of immune cell infiltration

A single-sample gene set enrichment analysis (ssGSEA) algorithm quantified the relative abundance of each gene in each sample. The enrichment scores calculated by the ssGSEA analysis represent the relative abundance of immune cell infiltration in each sample based on the gene expression markers in the immune cells. The calculation was conducted using the “GSVA” R package (39). The immune cell infiltration patterns of the two risk groups were visualized in a heatmap. In addition, the correlation of infiltration levels of different immune cells and the correlation between the levels of the key MRDEGs and infiltration levels of immune cells in the two risk groups were subject to Pearson correlation analysis under the threshold value of *p* < 0.05, and data visualization was conducted using the “ggplot2” R package.

The cell-type identification by estimating relative subsets of RNA transcripts (CIBERSORT) deconvolution algorithm is also applied to estimate the composition and abundance of different types of immune cells in a cell mixture (40). The expression matrix data in AMI, combined with the leukocyte signature matrix (LM22) feature gene matrix, were used to screen the immune cells with enrichment scores greater than zero. The comparison between the groups was visualized using stacked bar charts. Additionally, the correlation between infiltration levels of different immune cells, or between infiltration levels and the levels of the key MRDEGs in the two risk groups were subject to Pearson correlation analysis using the “ggplot2” R package to visualize the results.

### 2.9 Interaction network analysis of key MRDEGs

The starBase database (version 3.0, https://rnasysu.com/encori/) is a platform that displays the interactions between miRNA–ncRNA, miRNA–mRNA, ncRNA–RNA, and RNA–RNA based on deep mining of high-throughput sequencing data from the RNA–RNA interactome, CLIP-seq, and degradome sequencing (degradome-seq) (41). MiRNAs that could potentially bind to the key MRDEGs were screened using the starBase platform using the criterion of pancancerNum >8. In additional, we screened the potential RNA-binding proteins (RBPs) on the starBase platform using the criterion of clusterNum >10.

The CHIPBase database (version 3.0, https://rna.sysu.edu.cn/chipbase/) (42) shows combined base sequence matrixes and binding sites according to DNA-binding protein ChIP-seq data and predicts the transcriptional regulation of TFs on genes. The potential TFs that could bind to the key genes were searched for in the CHIPBase database using the criteria of >10 upstream and downstream samples.

The Comparative Toxicogenomics Database (CTD, http://ctdbase.org/) is a platform that links genes, chemicals, phenotypes, diseases, and known toxicological information (43). Drugs or small molecule compounds that could interact with the key genes were predicted using the CTD with the criterion of “Reference Count” >2.

The interaction networks between the key MRDEGs and miRNAs, RBPs, TFs, and drugs were finally visualized via Cytoscape software. The interaction among MRDEGs was also explored using the STRING database (https://cn.string-db.org/) (44), and the protein–protein interaction (PPI) network was constructed using Cytoscape software (version 3.9.1).

### 2.10 Spatial protein structure of key genes

The Alphafold platform (45) has proposed that, even without a homologous template, a protein’s structure can be predicted with atomic precision based on a calculation, most of the known proteins in Homo sapiens included. The protein structure of each predicted key MRDEG on the Alphafold2 website was downloaded and visualized.

### 2.11 Clinical sample collection

The blood samples were collected from 20 patients with an AMI and healthy control individuals. The samples were centrifuged at 3,000 rpm at 4°C for 10 min, and the plasma sample was stored at −80°C until use. The study was conducted following the principles in the Declaration of Helsinki, and informed consent was obtained from all participants.

### 2.12 Real-time quantitative polymerase chain reaction

Total RNA was extracted using Trizol reagent (Invitrogen, USA), and reverse transcribed into cDNA using a Script High Fidelity One Step RT-PCR Kit (Vazyme, China) following the manufacturer's instructions. A polymerase chain reaction (PCR) was then performed using SYBR Green Mix on an ABI 7500 Fast Real-Time PCR system (Applied Biosystems, USA). The relative expression of the genes was calculated using the 2^−ΔΔCt^ method with *GAPDH* as the internal control. The primer sequences were as follows: *AGPS*-F: 5′- CGGGTTCTCTCTGGCCATC-3′, *AGPS*-R: 5′-TCTTGCCGCTTCTTTGGGAT-3′; *CA2*-F: 5′-CAAACACAACGGACCTGAG-3′, *CA2*-R: 5′-AGTGTCGATGTCAACAGGG-3′; *CAT*-F: 5′-CTGTGAACTGTCCCTACCG-3′, *CAT*-R: 5′-TAATTTGGAGCACCACCCT-3′; *LTA4H*-F: 5′-CTCTACTCTCCTGGACTGC-3′, *LTA4H*-R: 5′-CATCTTCTTTGGCAGTAATCCA -3′; *MYO9B*-F: 5′-TTCCAAAGAACCTACTGGACTC-3′, *MYO9B*-R: 5′- CAGTAGGGACTTGGTGGTG-3′; *PRDX6*-F: 5′-GAGGACCATCTTGCCTGGAG-3′, *PRDX6*-R: 5′-AAACACCACACGAGCTGTCA-3′; *PYGB*-F: 5′-CCATGACAGGTTCAAGGTG-3′, *PYGB*-R: 5′-TTCTTGGTCCACTCCTTGG-3′; *SIRT3*-F: 5′-AGGGACGATTATTAAAGGTGGA-3′, *SIRT3*-R: 5′-TACATCCTGCAGGGAAAGC-3′; *TFEB*-F: 5′-AGCTCACAGATGCTGAGAG-3′, *TFEB*-R: 5′-TGTTGAACCTTCGTCTCCT-3′; *TOM1*-F: 5′-TCAACCTCATCCAGTCCTG-3′, *TOM1*-R: 5′-GCAGGTCCTCATAGATGGT-3′; *UBA52*-F: 5′-AAACCATCACCCTTGAGGT-3′, *UBA52*-R: 5′-GTGGGATACCCTCCTTGTC-3′; *UBB*-F: 5′-AGATGGCCGTACTCTTTCTG-3′, *UBB*-R: 5′-TGCATACCACCTCTCAGAC-3′; *GAPDH*-F: 5′-TCAAGATCATCAGCAATGCC-3′, *GAPDH*-R: 5′-CGATACCAAAGTTGTCATGGA-3′.

### 2.13 Statistical analysis

R software (V 4.2.3) was used for data processing and analyses. Continuous variables are presented as the mean ± standard deviation and compared by the Wilcoxon rank sum test between the two groups, while the independent Student's *t*-test evaluated the statistical differences between the normally distributed variables. The difference in multiple groups was compared using the Kruskal–Wallis test. The comparison between categorical variables was subject to the chi-square test or Fisher's exact test. Spearman’s or Pearson’s correlation analyses were used for the correlation analyses. A value of *p* < 0.05 was regarded as the threshold value.

## 3 Results

### 3.1 Screening of MRDEGs in AMI

**Figure 2:**
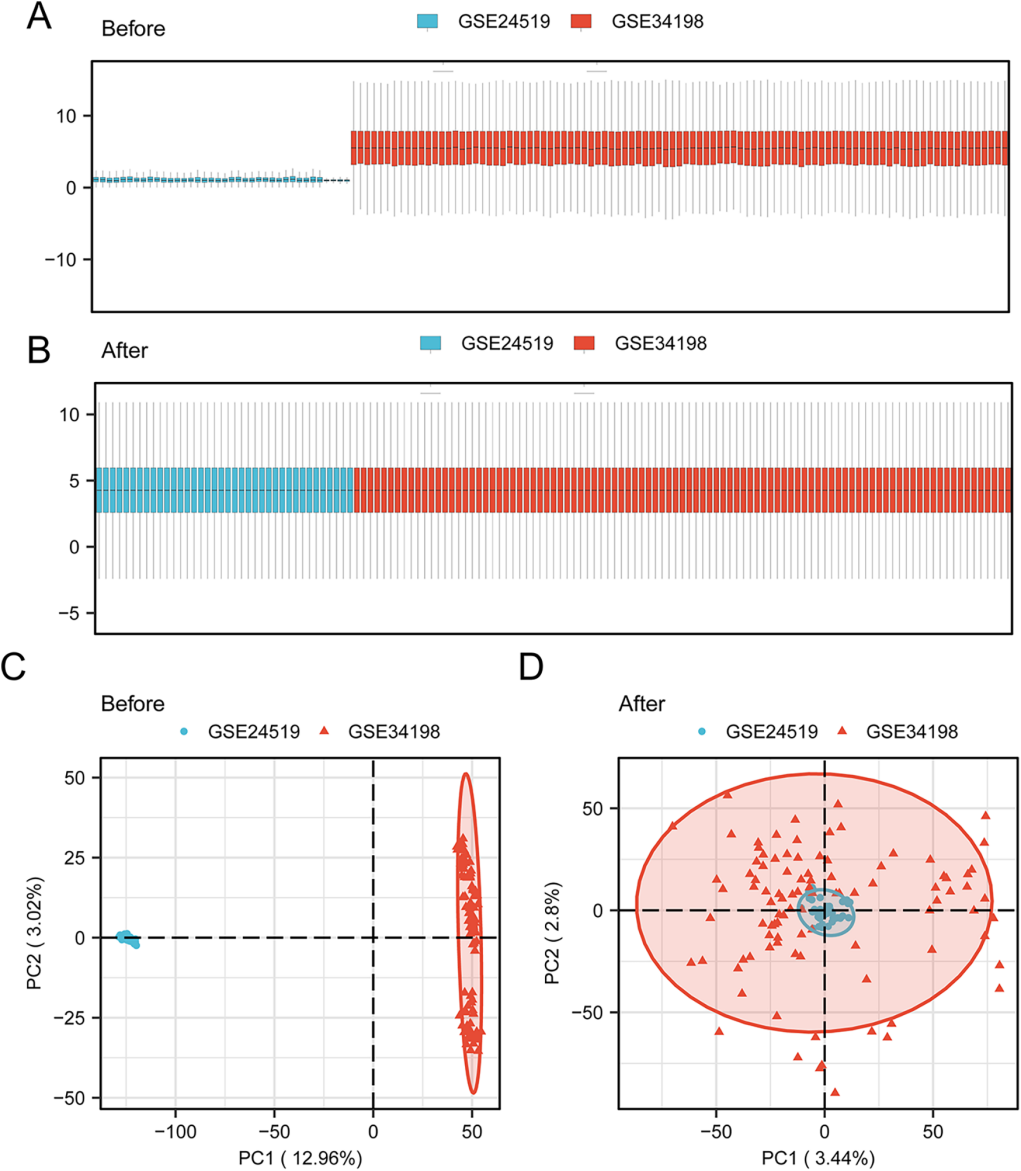
Data collection and pre-processing. Box plot of the AMI datasets **(A)** before correction and **(B)** after collection. PCA plot of the AMI datasets **(C)** before correction and **(D)** after collection. AMI, acute myocardial infarction; PCA, principal component analysis.

Human AMI datasets (GSE24519 and GSE34198) were combined and the batch effects were removed with the “sva” R package. As revealed by the distribution boxplot and principal component analysis, the batch effects of the samples in the two datasets were eliminated (Figures 2A–D).

**Figure 3:**
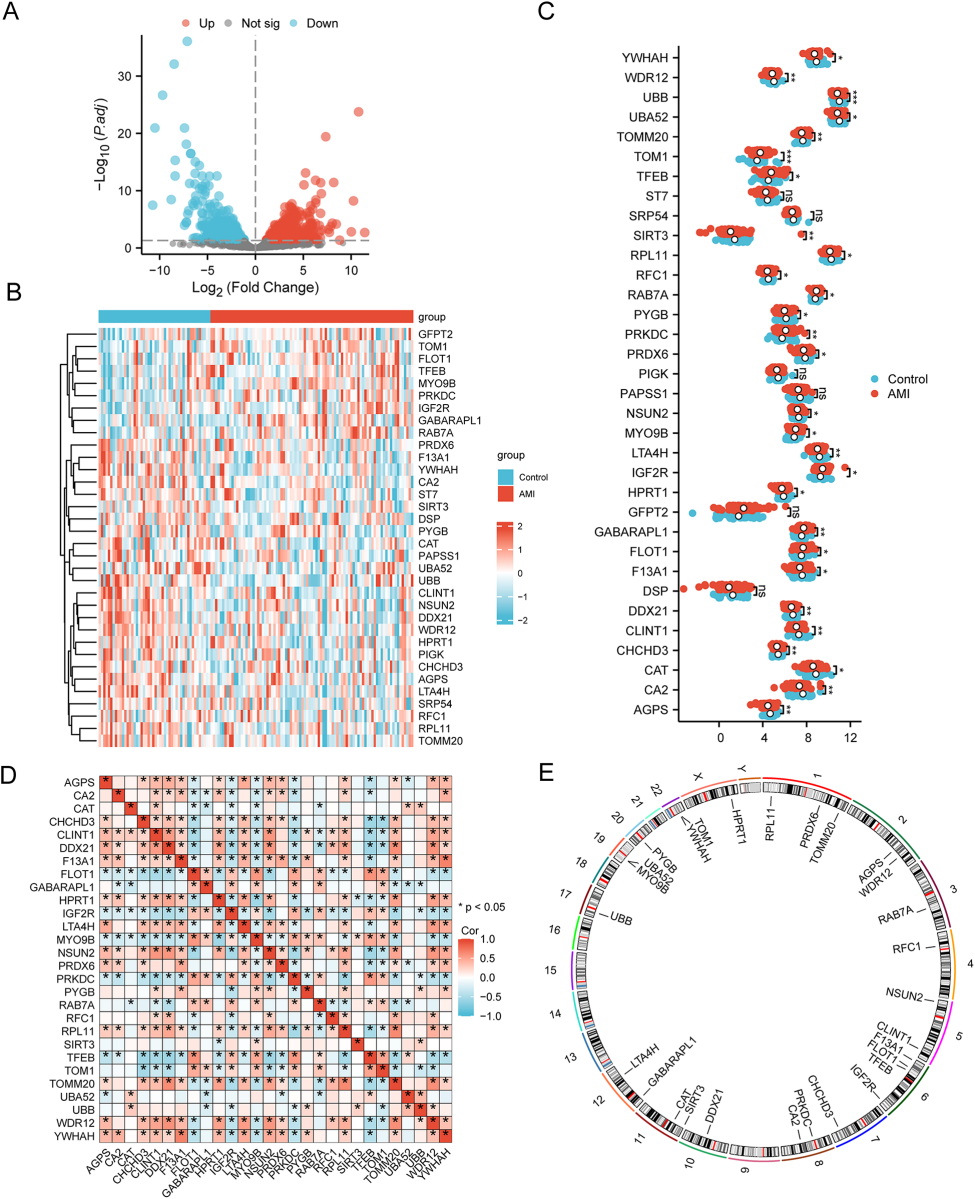
Selection of MRDEGs in AMI. **(A)** Volcano plot of the DEGs between the AMI group and control group. **(B)** Heatmap of 34 MRDEGs between the AMI group and control group. **(C)** Group comparison of MRDEGs between the AMI group and control group. **(D)** Correlation heatmap of the 28 MRDEGs using Spearman’s correlation analysis. **(E)** Chromosomal mapping of the 28 MRDEGs. AMI, acute myocardial infarction; DEGs, differentially expressed genes; MRDEGs, mitophagy-related differentially expressed genes. *p < 0.05; **p < 0.01; ***p < 0.001.

The DEGs in the AMI samples compared with normal samples were screened using the criteria of |logFC| >0 and *p* <0.05. A total of 352 DEGs in AMI were obtained, including 192 upregulated DEGs and 160 downregulated DEGs (Figure 3A). These DEGs were intersected with the MRGs obtained from GeneCards and previous literature, and 34 MRDEGs were finally obtained. These were *AGPS, CA2, CAT, CHCHD3, CLINT1, DDX21, DSP, F13A1, FLOT1, GABARAPL1, GFPT2, HPRT1, IGF2R, LTA4H, MYO9B, NSUN2, PAPSS1, PIGK, PRDX6, PRKDC, PYGB, RAB7A, RFC1, RPL11, SIRT3, SRP54, ST7, TFEB, TOM1, TOMM20, UBA52, UBB, WDR12,* and *YWHAH*. The expression profiles of these MRDEGs in the AMI and control samples are shown in a heatmap (Figure 3B). A group comparison of 34 MRDEGs between AMI and control samples was then conducted, and 28 MRDEGs were shown to be differentially expressed in AMI with statistical significance, namely, *AGPS, CA2, CAT, CHCHD3, CLINT1, DDX21, F13A1, FLOT1, GABARAPL1, HPRT1, IGF2R, LTA4H, MYO9B, NSUN2, PRDX6, PRKDC, PYGB, RAB7A, RFC1, RPL11, SIRT3, TFEB, TOM1, TOMM20, UBA52, UBB, WDR12,* and *YWHAH*. *TOM1* and *UBB* were extremely differentially expressed in AMI (*p* < 0.001) (Figure 3C). We then analyzed the correlation between the 28 MRDEGs using Spearman’s correlation analysis (Figure 3D). The locations of the 28 MRDEGs on human chromosomes were subsequently analyzed and annotated accordingly (Figure 3E). These MRDEGs were distributed on most chromosomes, among which chromosome 6 was the most commonly distributed with four MRDEGs.

### 3.2 Functional enrichment analyses of MRDEGs

**Figure 4:**
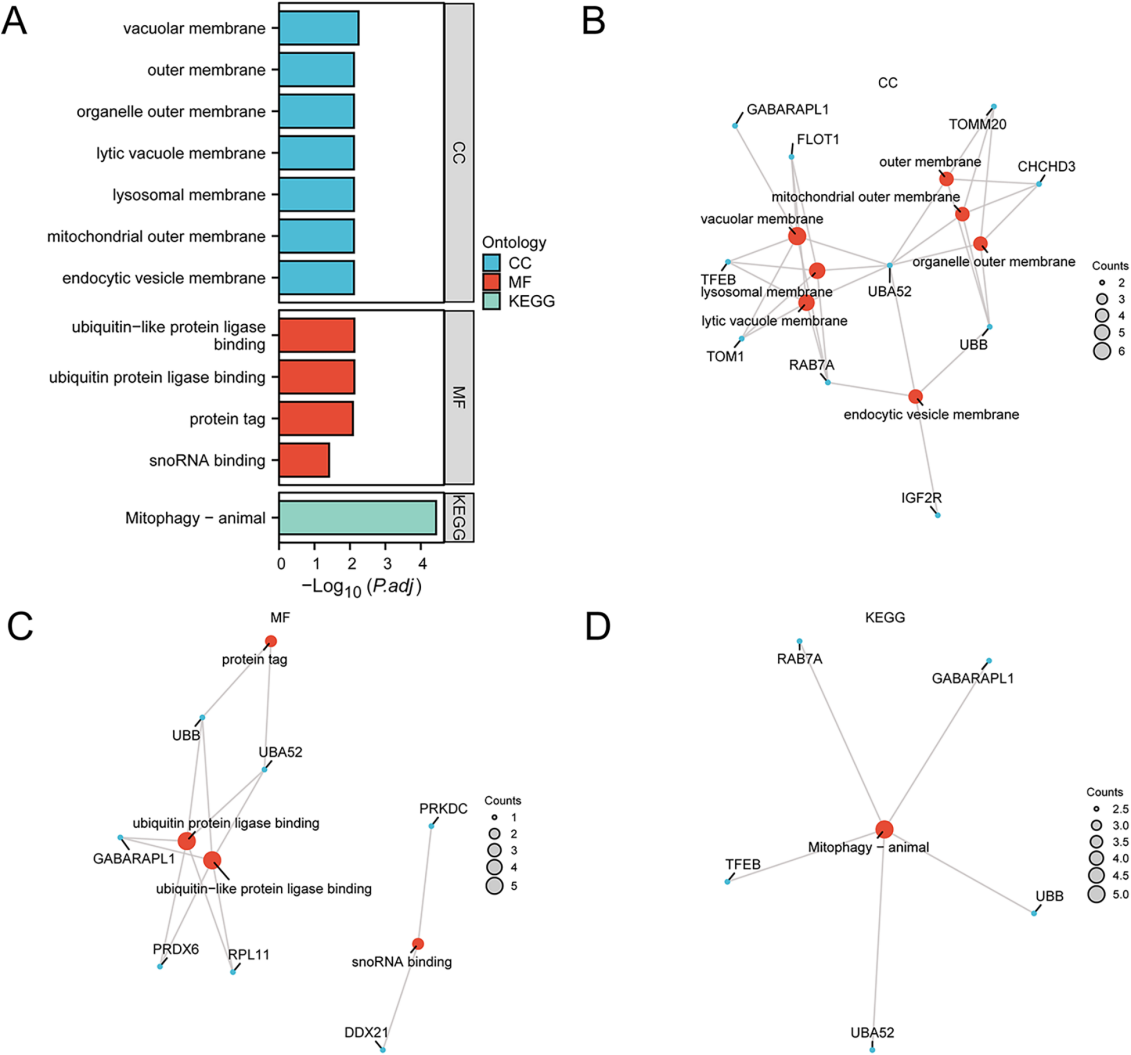
Functional enrichment analyses of the MRDEGs. **(A)** GO and KEGG enrichment analyses of the MRDEGs depicted in a bar graph. **(B**–**D)** Network diagrams of GO and KEGG enrichment analyses of MRDEGs, respectively. GO, Gene Ontology; KEGG, Kyoto Encyclopedia of Genes and Genomes; MRDEGs, mitophagy-related differentially expressed genes.

To characterize the 28 MRDEGs, GO and KEGG enrichment analyses were performed with *p*.adj <0.05 and FDR <0.05 (Figures 4A–D and Supplementary Table 2). According to the GO enrichment analysis, the MRDEGs were significantly enriched in various biological membranes such as mitochondrial outer membrane, lysosomal membrane, organelle outer membrane, and others in CC, and significantly enriched in ubiquitin or ubiquitin-like protein ligase binding, protein tag, and snoRNA binding in MF. The BP results were not shown due to a lack of statistical significance. The KEGG analyses showed that the MRDEGs were enriched in mitophagy-related pathways.

**Figure 5:**
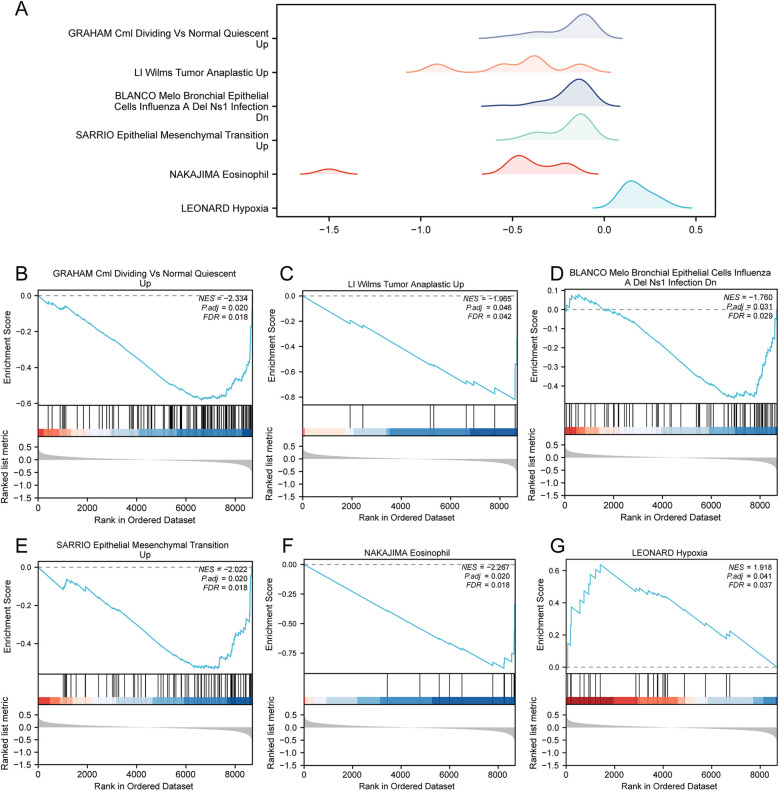
GSEA in the AMI datasets. **(A)** GSEA of the combined datasets between the AMI and control samples, including six main biological characteristics, namely **(B)** GRAHAM Cml dividing vs normal quiescent, **(C)** LI Wilms Tumor Anaplastic, **(D)** BLANCO Melo Bronchial Epithelial Cells Influenza A Del Ns1 Infection, **(E)** SARRIO Epithelial Mesenchymal Transition, **(F)** NAKAJIMA Eosinophil, and **(G)** LEONARD Hypoxia and other pathways. GSEA, gene set enrichment analysis; AMI, acute myocardial infarction.

GSEA was applied to determine the difference in functional enrichment pathways between the control and AMI groups (Figure 5A and Supplementary Table 3). The results showed that the GRAHAM Cml Dividing VS Normal Quiescent (Figure 5B), LI Wilms Tumor Anaplastic (Figure 5C), BLANCO Melo Bronchial Epithelial Cells Influenza A Del Ns1 Infection (Figure 5D), SARRIO Epithelial Mesenchymal Transition (Figure 5E), NAKAJIMA Eosinophil (Figure 5F), and LEONARD Hypoxia (Figure 5G) pathways were differentially enriched between the AMI and control groups.

### 3.3 Construction of the LASSO risk model and the selection of the key genes

**Figure 6:**
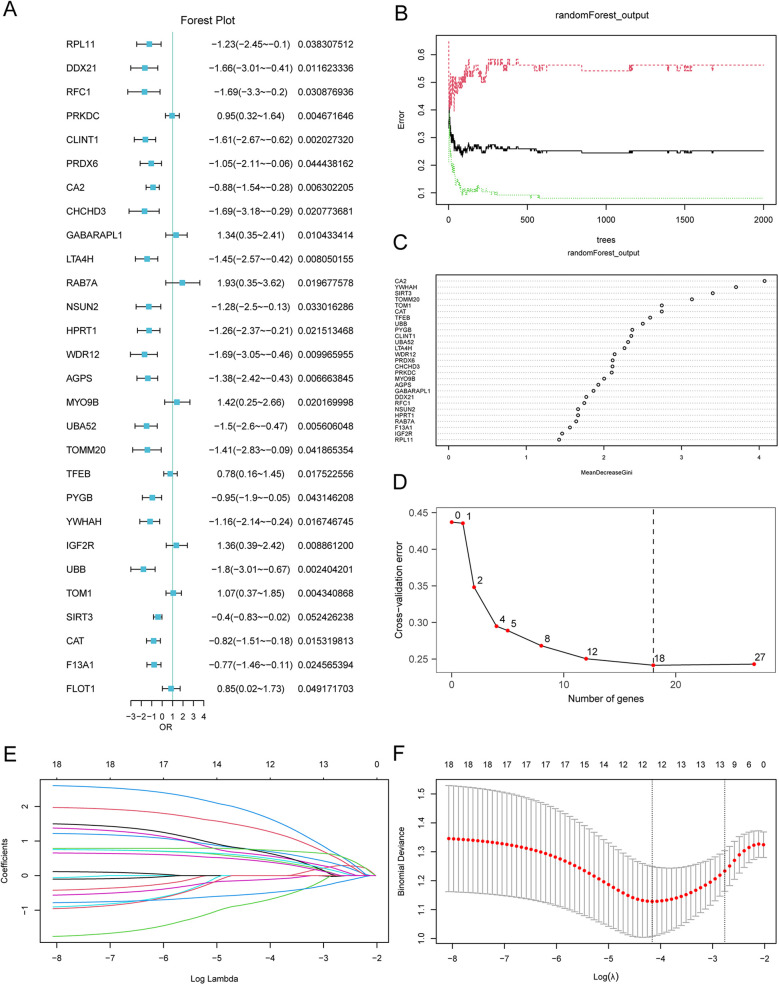
LASSO risk model construction and screening of key genes. **(A)** Forest plot of the 28 MRDEGs in the logistic regression model. **(B)** Plot of model training error using the random forest algorithm. **(C)** Scatterplot of the random forest model based on analysis of the MeanDecreaseGini. **(D)** Cross-validation error plot. **(E)** Diagnostic model plot of the LASSO regression model. **(F)** Variable trajectory plot of the LASSO regression model. LASSO, least absolute shrinkage and selection operator. MRDEGs, mitophagy-related differentially expressed genes.

To construct an AMI risk model, we conducted a logistic regression analysis with *p* <0.05 as the cutoff value, and the expressions of the MRDEGs in AMI are shown in Figure 6A. A total of 27 key MRDEGs were screened and subjected to RF analysis (Figure 6B). The error curve of the decision tree was drawn with a seed of 150 and the number of decision trees as 2,000. The results showed that the error had leveled off when the number of decision trees was 2,000. MeanDecreaseGini represents the importance of the genes for our grouping (Figure 6C). Next, we performed ten-fold cross-validation five times to select the number of genes (Figure 6D). We found that when the number of genes was 18, the error of the model was relatively small and tended to be stable with the increase in the number of genes. In total, 18 MRDEGs were indicated to be crucial for AMI diagnosis, namely *AGPS, CA2, CAT, CHCHD3, CLINT1, LTA4H, MYO9B, PRDX6, PRKDC, PYGB, SIRT3, TFEB, TOM1, TOMM20, UBA52, UBB, WDR12,* and *YWHAH*.

Furthermore, we conducted the LASSO regression analyses to construct the LASSO risk model using the 18 selected MRDEGs, and analysis data were output by drawing a diagram for the LASSO regression model and a LASSO variable trajectory diagram (Figures 6E,F). A total of 12 MRDEGs were included in the LASSO risk model, namely *AGPS, CA2, CAT, LTA4H, MYO9B, PRDX6, PYGB, SIRT3, TFEB, TOM1, UBA52,* and *UBB*. These 12 MRDEGs were selected as the key genes for our follow-up study. In addition, we also divided the AMI patients to low- and high-risk groups on the basis of the median risk score. The calculation of risk score was conducted as follows:

RiskScore=1.331×AGPS+0.727×CA2+0.416×CAT+0.596×LTA4H+−0.743×MYO9B+0.475×PRDX6+0.69×PYGB+0.527×SIRT3+−0.119×TFEB+−0.516×TOM1+1.739×UBA52+0.748×UBB

### 3.4 Performance of the key MRDEGs for AMI diagnosis

**Figure 7:**
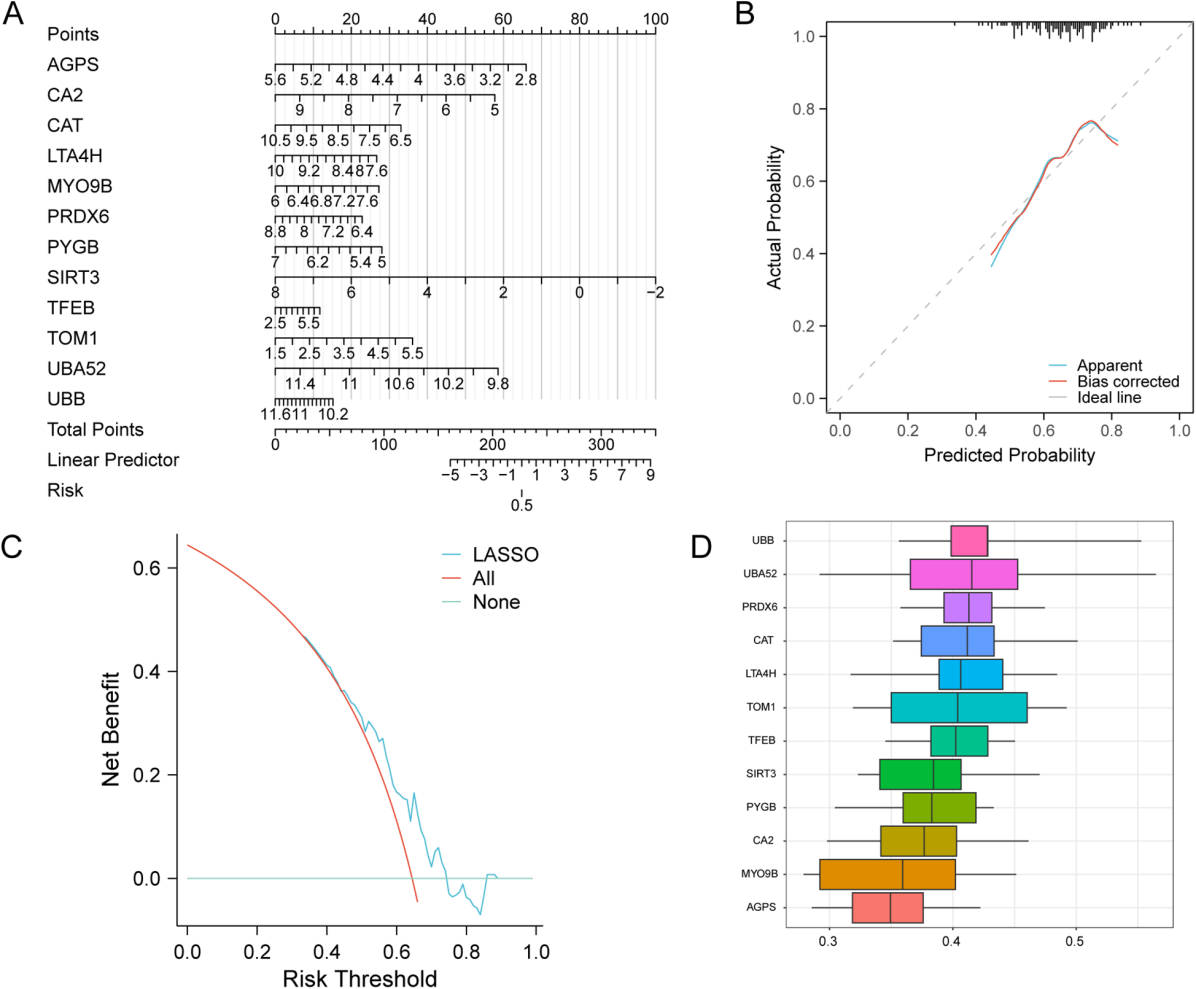
Diagnostic ability of the key genes in AMI. **(A)** A nomogram of the key genes in the AMI LASSO risk diagnosis model. **(B)** Calibration curve of the key genes in the LASSO risk diagnosis model. **(C)** DCA plot of the key genes of the LASSO risk diagnosis model. **(D)** Functional similarity analysis of the key genes. LASSO, least absolute shrinkage and selection operator; AMI, acute myocardial infarction.

To evaluate the diagnostic performance of the key genes selected by the LASSO risk model for AMI, a logistic regression diagnostic model and a nomogram were used to reveal the contributions of the expression of the 12 selected MRDEGs to AMI (Figure 7A). The results showed that *SIRT3* expression level significantly benefits AMI diagnosis, while *TFEB* showed the lowest contribution to AMI diagnosis compared with the other key genes. On the calibration curve, the solid line showed the fitting of the optimal theoretical probability and the dashed line showed the evaluation of probability predicted by the model in AMI cases, and we found a favorable predictive effect of the model when compared to the actual results (Figure 7B). Subsequently, the decision curve analysis evaluated the efficacy of this model for AMI diagnosis, which verified the benefit of the model for AMI diagnosis (Figure 7C). We then performed the functional similarity analyses of the 12 key MRDEGs, among which *UBB* showed the highest functional similarity compared with other MRDEGs, which suggested that *UBB* might play a key role in AMI (Figure 7D).

### 3.5 Gene set variation analysis based on key genes

**Figure 8:**
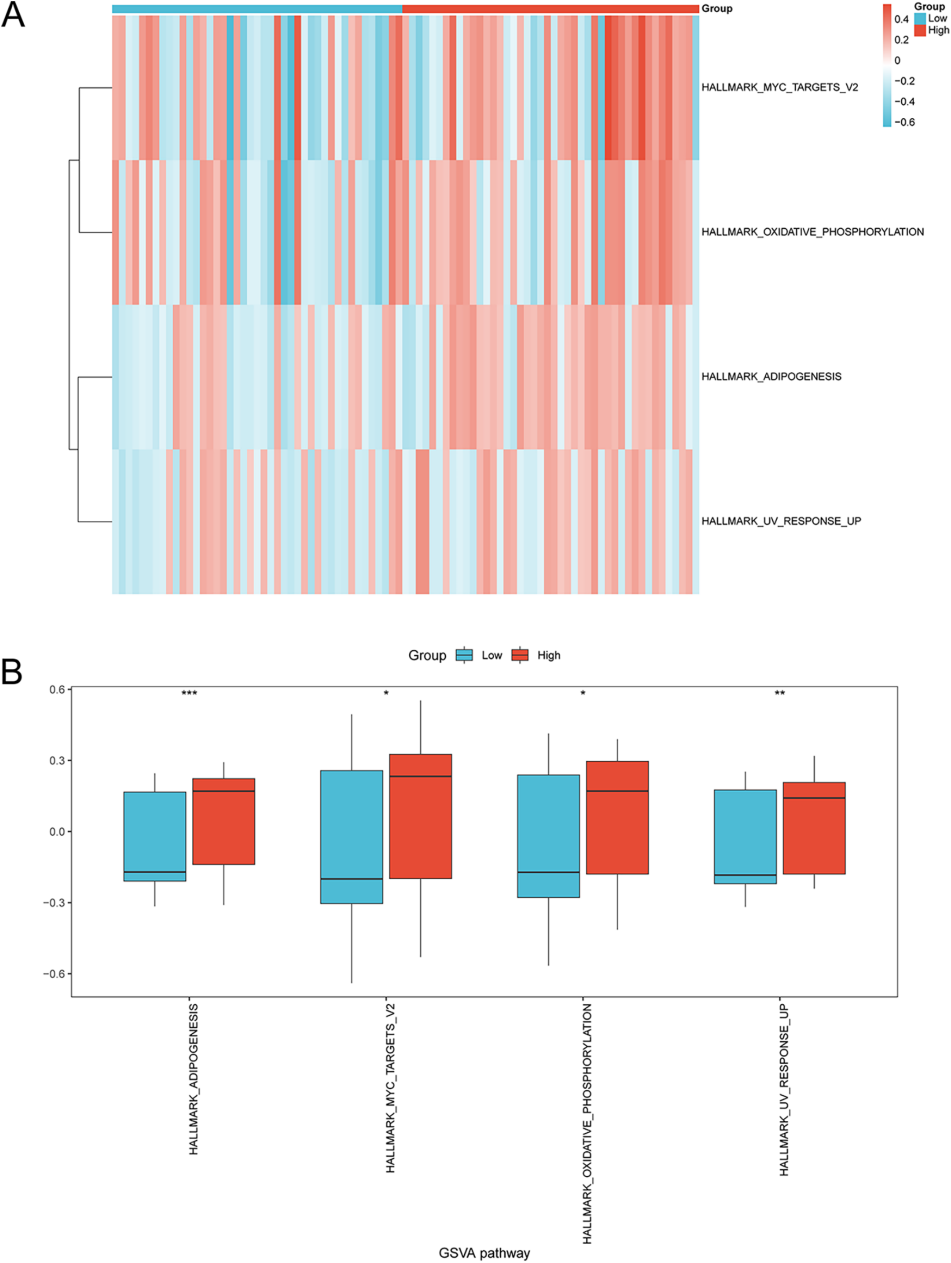
GSVA of enriched pathways in the high-risk and low-risk groups. **(A)** Heatmap and **(B)** group comparison diagram showing the difference in enriched pathways in the high- and low-risk groups via GSVA. *p < 0.05, **p < 0.01, ***p < 0.001. GSVA, gene set variation analysis; AMI, acute myocardial infarction.

GSVA was used to explore the difference of the h.all.v7.4.symbols.gmt gene set and four pathways exhibited differential distribution between the two risk groups, including adipogenesis, Myc targets, oxidative phosphorylation, and UV response (Figure 8A and Supplementary Table 4). The group comparison map showed the upregulation of these pathways in the high-risk group compared to the low-risk group (Figure 8B).

### 3.6 Immune infiltration analysis

**Figure 9:**
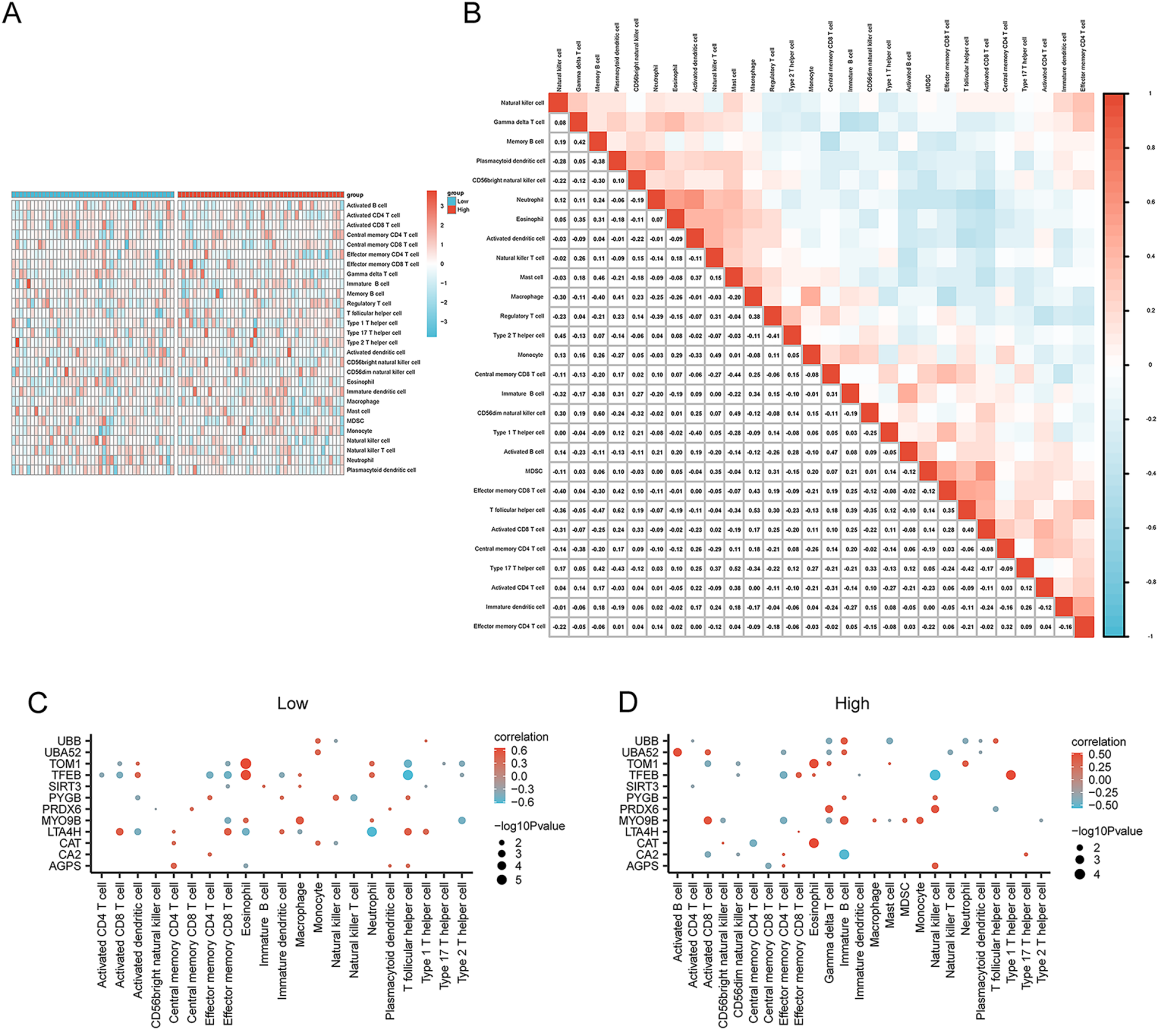
Analysis of immune cell infiltration in the high- and low-risk groups via ssGSEA. **(A)** Heatmap showing the difference in immune infiltration between the high- and low-risk groups. **(B)** Correlation analysis of immune cell infiltration abundance in the AMI samples. Correlation between the infiltration levels of immune cells and the expression of key genes in the **(C)** low-risk group and **(D)** the high-risk group. *p < 0.05; **p < 0.01; ***p < 0.001. AMI, acute myocardial infarction; ssGSEA, single-sample GSEA.

ssGSEA was conducted to explore the immune infiltration patterns in the two risk groups in AMI by calculating the infiltrating abundance of 28 types of immune cells. The results revealed that the infiltration levels of immune cells such as gamma delta T cells and natural killer (NK) cells showed a lower proportion in the high-risk group compared with the low-risk group (Figure 9A). The correlation between the infiltration levels of different immune cells in the AMI samples was then analyzed and presented. For example, the results showed that the infiltration levels of natural killer cells were positively correlated with type 2 T helper cells (Th2 cells) and CD56dim natural killer cells, and negatively correlated with macrophages and immature B cells. The abundance of gamma delta T cells was positively correlated with memory B cells and eosinophils, and negatively correlated with central memory CD4 T cells. The abundance of memory B cells was positively correlated with eosinophils, Mast cells, and CD56dim natural killer cells, and negatively correlated with plasmacytoid dendritic cells, CD56bright natural killer cells, macrophages, immature B cells, and effector memory CD8 T cells (Figure 9B). Furthermore, we found the expression of the 12 selected key genes was significantly correlated with the infiltration level of 21 or 25 types of immune cells excluding gamma delta T cells, CD56dim natural killer cells, myeloid-derived suppressor cells (MDSCs), and activated B cells in the low-risk group, and including all 25 types of immune cells in the high-risk group. For example, the immune cell infiltration levels of activated CD4 T cells were negatively correlated with *TFEB* expression in the low-risk AMI group (*p* < 0.05) (Figure 9C). In the high-risk AMI group, for example, the infiltration level of activated B cells was positively correlated with the expression of *UBA52* (Figure 9D).

**Figure 10:**
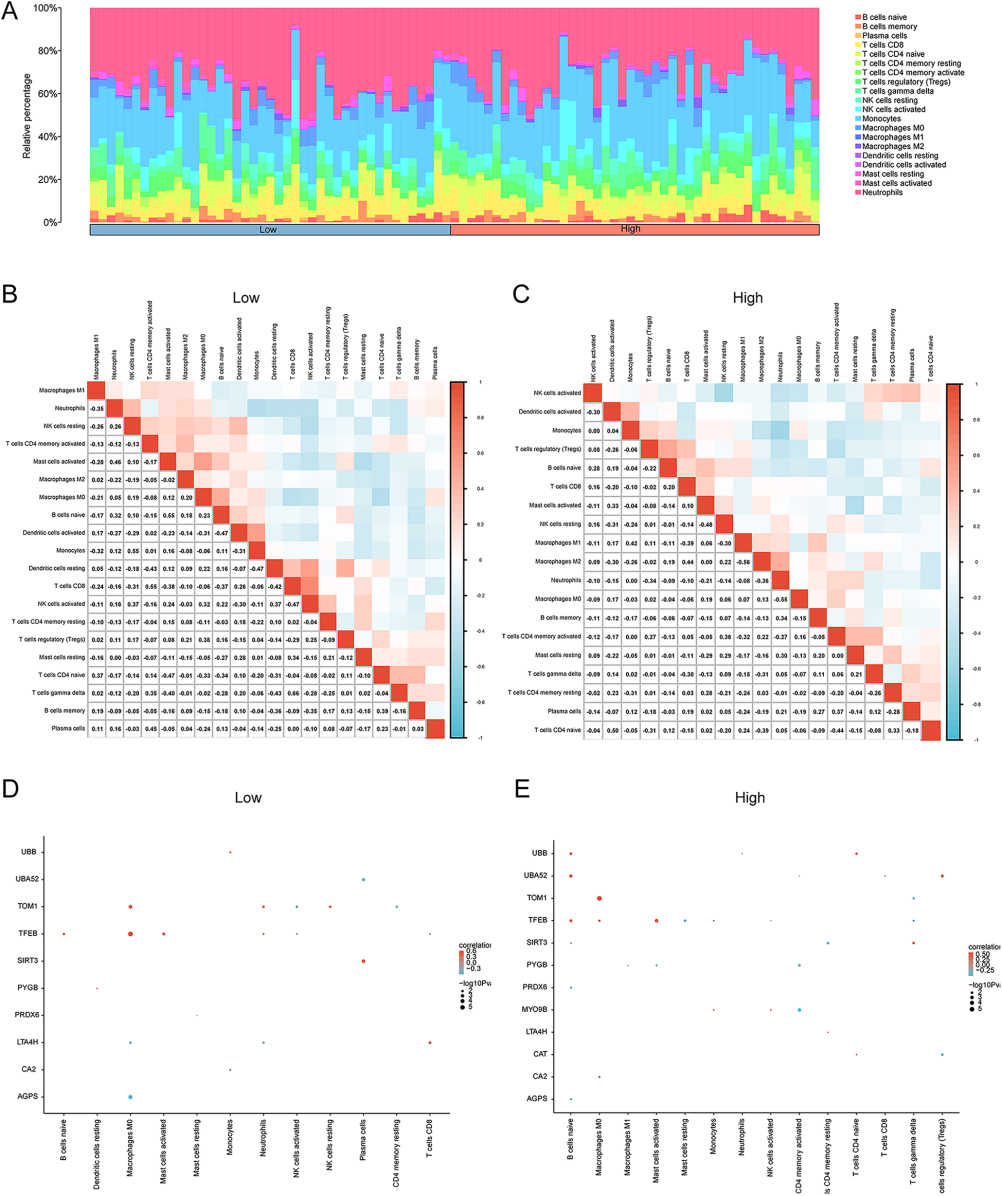
Immune cell infiltration analysis of the AMI dataset samples via CIBERSORT. (**A**) Stacking diagram of immune cell infiltration between the high- and low-risk groups. The correlation heatmap illustrating the correlations among infiltrating immune cells in (**B**) the low-risk group and (**C**) the high-risk group. Linear regression analysis revealing a significant correlation between key genes and infiltrating immune cells in the **(D)** low- and **(E)** high-risk groups. *p < 0.05, **p < 0.01, ***p < 0.001. AMI, acute myocardial infarction.

Furthermore, CIBERSORT was also applied to calculate the infiltration levels of immune cells in the two risk groups. We found the enrichment of 20 immune cells (abundance >0) including B cells, T cells, plasma cells, NK cells, and others. As shown in Figure 10A, neutrophils, macrophages, NK cells, and B cells were enriched in the AMI samples. Moreover, the correlation heatmap visualized the relationships between the infiltrating immune cells in the low- and high-risk groups (Figures 10B,C). Linear regression analysis demonstrated a remarkable correlation between key genes and infiltrating immune cells in the low- and high-risk groups (Figures 10D,E).

### 3.7 Analysis of interaction networks for key MRDEGs

**Figure 11:**
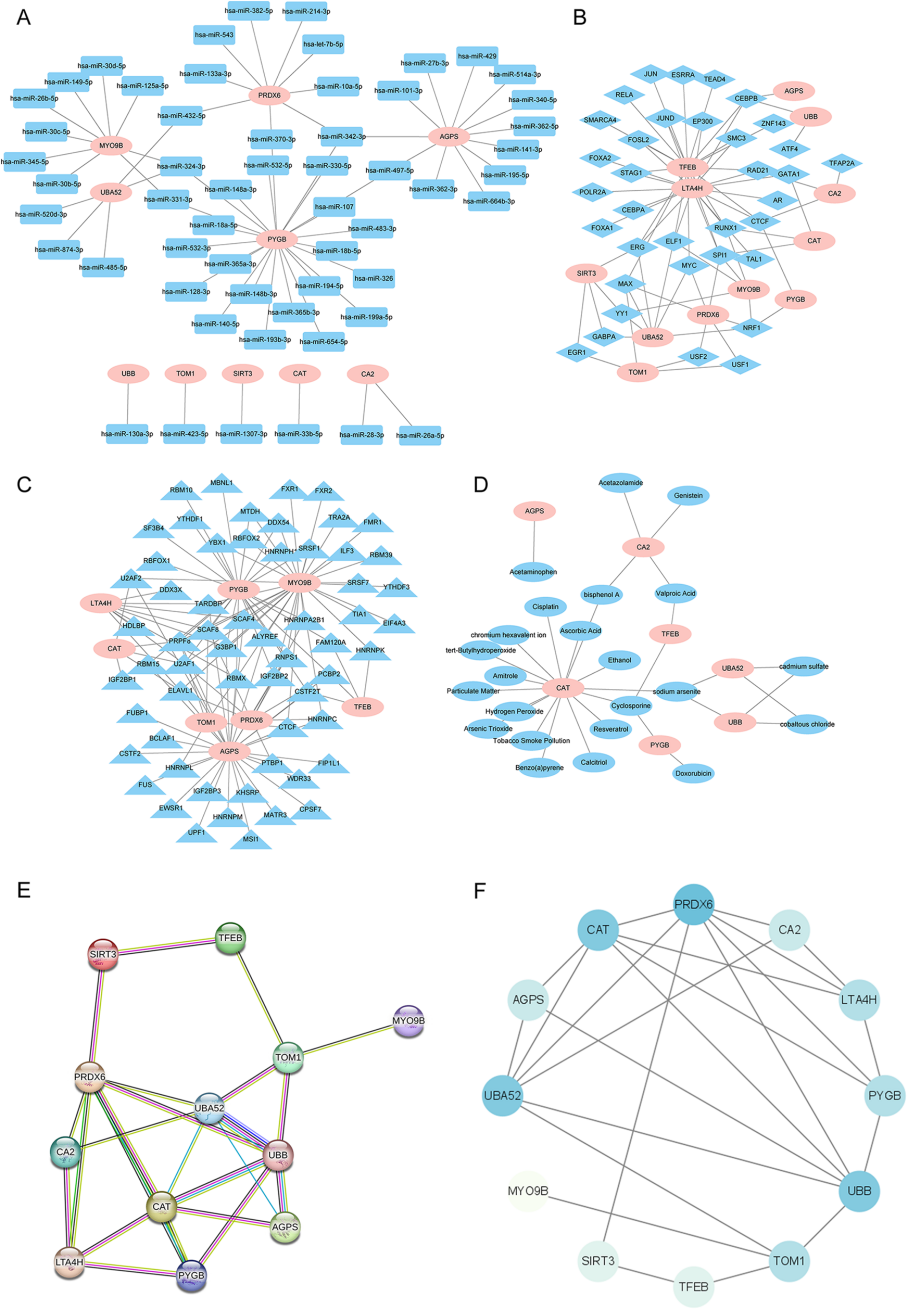
Interaction network analysis of the key genes. (**A**) The mRNA–miRNA interaction network of the key genes and potential binding miRNAs predicted by the starBase database. (**B**) The mRNA–TF interaction network of the key genes and potential TFs predicted by the CHIPBase database. (**C**) The mRNA–RBP interaction network of the key genes and potential RBPs predicted by the starBase database. (**D**) The mRNA–drug interaction networks of the key genes and potentially interacting molecular compounds as predicted by the CTD database. (**E,F**) The PPI network of the 12 key genes was constructed using the STRING database and Cytoscape software. miRNA, microRNA; TF, transcription factor; RBP, RNA-binding protein; CTD, Comparative Toxicogenomics Database; PPI, protein–protein interaction.

We further delved into the potential regulatory mechanisms of the 12 key MRDEGs. The starBase database predicted the miRNAs that could possibly bind to the 12 MRDEGs. The mRNA–miRNA interaction networks included 10 key genes (*AGPS, CA2, CAT, MYO9B, PRDX6, PYGB, SIRT3, TOM1, UBA52,* and *UBB*) and 56 miRNAs, and 64 pairs of mRNA–miRNA interaction relationships were contained in the network (Figure 11A). CHIPBase database was used to search the TFs that could potentially bind to the 12 key genes, and 35 TFs were screened, with a total of 75 TF-mRNA interaction pairs (Figure 11B). In addition, the starBase platform predicted the RBPs that could bind to the key MRDEGs. The RBP–mRNA interaction network was composed of eight key genes (*AGPS, CAT, LTA4H, MYO9B, PRDX6,* and *PYGB*) and 60 RBPs, with 119 mRNA–RBP interaction pairs (Figure 11C). We then predicted the drugs or small molecule compounds with possible interactions with the 12 key MRDEGs using the CTD database. The drug–mRNA interaction network was constructed, which contained seven key genes (*AGPS, CA2, CAT, PYGB, TFEB, UBA52,* and *UBB*) and 23 molecules, with a total of 32 drug–mRNA interaction relationships (Figure 11D). Finally, the interaction among the 12 key genes was explored and a PPI network was constructed, and genes such as *PRDX6*, *CAT*, *UBA52,* and *UBB* showed close associations with other genes in the interaction network (Figures 11E,F).

### 3.8 Spatial protein structures of 12 MRDEGs

**Figure 12:**
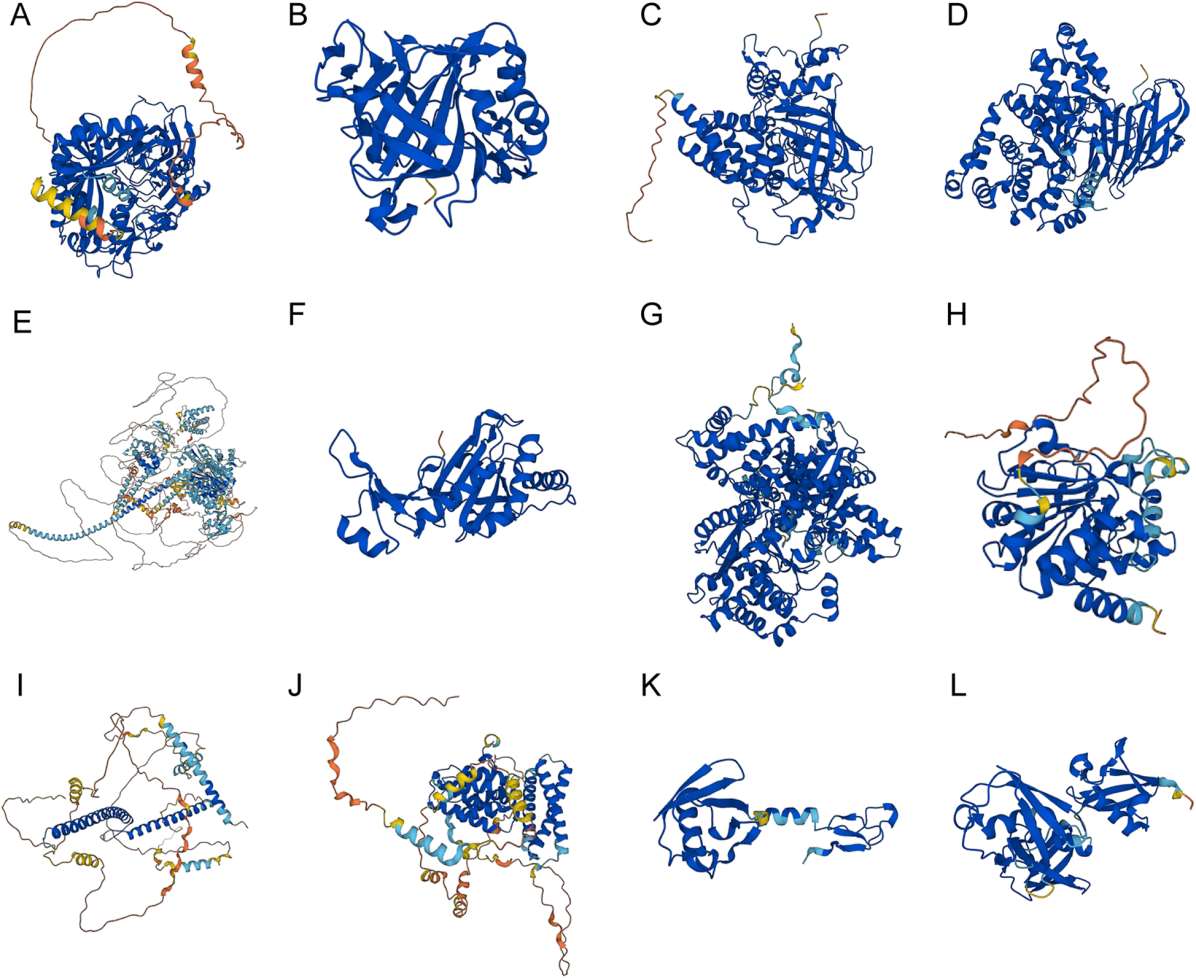
The spatial protein structures of the key genes. Protein structures of (**A**) *AGPS*, (**B**) *CA2*, (**C**) *CAT*, (**D**) *LTA4H*, (**E**) *MYO9B*, (**F**) *PRDX6*, (**G**) *PYGB*, (**H**) *SIRT3*, (**I**) *TFEB*, (**J**) *TOM1*, (**K**) *UBA52*, (**L**) and *UBB* on the AlphaFold website.

The protein structures of the 12 key genes were analyzed respectively, and visualized in Figures 12A–L. The confidence score per residue (pLDDT) on the AlphaFold platform is between 0 and 100. Regions in red were below 50 pLDDT and might be isolated unstructured regions, with very low model confidence; while regions with a pLDDT between 50 and 70 were marked yellow, and indicated the low confidence of model; regions with a pLDDT between 70 and 90 were in light blue, and model confidence was normal. A pLDDT above 90 (marked in blue) indicated very high model confidence. The visualization of the spatial protein structures of these key genes may provide clues for exploring their biological function.

### 3.9 Validation of the expression of the 12 MRDEGs in AMI

**Figure 13:**
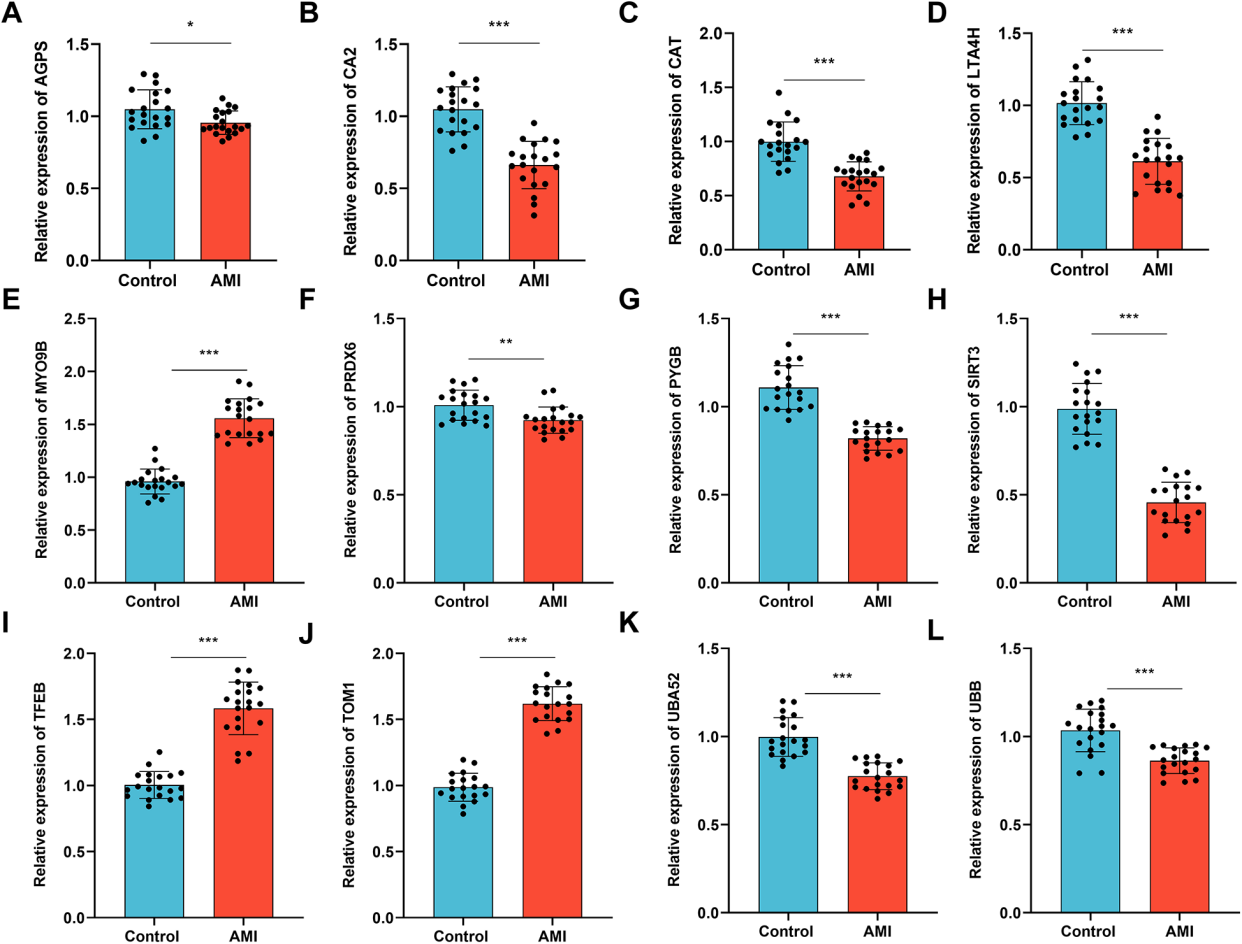
Validation of the expression of the key genes in the patients with an AMI and healthy controls. RT-qPCR was performed to examine the mRNA levels of (**A**) *AGPS*, (**B**) *CA2*, (**C**) *CAT*, (**D**) *LTA4H*, (**E**) *MYO9B*, (**F**) *PRDX6*, (**G**) *PYGB*, (**H**) *SIRT3*, (**I**) *TFEB*, (**J**) *TOM1*, (**K**) *UBA52*, (**L**) and *UBB* in plasma samples collected from patients with an AMI (*n* = 20) and healthy individuals (*n* = 20). Data are shown as the mean ± SD from three independent experiments. *p < 0.05; **p < 0.01; ***p < 0.001, unpaired *t*-test.

Furthermore, we investigated the expression of the 12 key MRDEGs in the plasma samples of patients with an AMI (*n* = 20) and healthy control individuals (*n* = 20). The results of the RT-qPCR analysis showed that the mRNA expression of *AGPS, CA2, CAT, LTA4H, PRDX6, PYGB, SIRT3, UBA52,* and *UBB* was downregulated in the AMI group compared with the controls, while the mRNA levels of *MYO9B, TFEB,* and *TOM1* were upregulated in the patients with an AMI relative to the controls (Figures 13A–L).

## 4 Discussion

Cardiovascular diseases lead to an estimated 17.9 million deaths annually across the world, and more than four out of five cardiovascular deaths are caused by an AMI and stroke (46). Despite the improved prognosis due to advances in anticoagulation, antiplatelet, thrombolytic, and interventional therapies, the clinical outcomes are negatively affected by complications such as cardiac ischemia/reperfusion and other pathologies, and some patients still progress to heart failure. Cardiac troponin I (cTnI) and cardiac troponin T (cTnT) are commonly used diagnostic biomarkers for AMI. However, their levels can be elevated in some other diseases, which limits their diagnostic specificity (47). Therefore, understanding AMI heterogeneity and exploring promising novel biomarkers are of vital significance to improve the diagnosis for timely treatment of patients with an AMI.

AMI has been revealed to cause mitochondrial Ca^2+^ overload, oxidative stress, and induce mitochondrial dysfunction and the death of cardiomyocytes (10). Increasing evidence has shown that mitophagy, as a response to acute tissue stress, is critically involved in immunity and the pathogenesis of AMI (17). Mitophagy can play a protective role against AMI by removing damaged mitochondria and maintaining mitochondrial and myocardial homeostasis (48). A recent study has identified five mitophagy-related hub genes (*SQSTM1*, *UBC*, *MFN2*, *ATG5*, and *TOMM20*) for AMI diagnosis using RandomForest and support vector machine-recursive feature elimination (SVM-RFE) algorithms (49). Wu et al. explored ferroptosis-related genes in AMI and screened seven biomarkers for AMI diagnosis based on ROC curves and experimental validation, including *ALOX5*, *CAMKK2*, *KDM6B*, *LAMP2*, *PTEN*, *PTGS2*, and *ULK1* (50). In this study, we explored mitophagy-related key genes involved in AMI. We screened the DEGs in AMI using AMI datasets and intersected the DEGs with mitophagy-related genes to obtain MRDEGs in AMI. Functional enrichment analyses were then performed for the selected MRDEGs to identify the biological functions and enriched pathways. Through machine learning methods including logistic regression analysis, RandomForest analysis, and LASSO analysis, we identified 12 key genes for AMI diagnosis. The diagnostic efficacy of the model in AMI was validated by a nomogram. Furthermore, immune infiltration analysis was conducted to reveal the immune landscape in two risk groups of AMI patients. Additionally, we established interaction networks of the key genes and analyzed their spatial protein structures. The findings of our study might deepen our understanding of the role of mitophagy in AMI pathogenesis and provide novel mitophagy-related biomarkers for AMI diagnosis.

In our study, a total of 12 key genes (*AGPS, CA2, CAT, LTA4H, MYO9B, PRDX6, PYGB, SIRT3, TFEB, TOM1, UBA52,* and *UBB*) were selected based on the logistic regression analysis, random forest, and LASSO algorithms. Some of the key genes have previously been reported as protective or risk factors for patients with an AMI, while their value as diagnostic biomarkers is rarely reported. For example, *CA2* is a critical target for the treatment of myocardial infarction (51). *LTA4H* is an independent risk factor for sudden cardiac death (52). *SIRT3* upregulation is related to autophagy activation and is a drug target to attenuate mitochondrial dysfunction in myocardial infarction (53). *TFEB* overexpression was demonstrated to alleviate cardiac remodeling following infarction (54). *PRDX6* protects from ventricular remodeling post-infarction (55). In our study, we identified the differential expression of these key genes compared with the healthy controls, which was consistent with the previous studies. The involvement of genes such as *AGPS, MYO9B, PYGB, TOM1, UBA52,* and *UBB* in myocardial infarction is first reported in this study. A LASSO risk model was generated, and AMI samples were divided into high- and low-risk groups. The diagnosis value of the 12 MRDEGs was evaluated using a nomogram, and the diagnostic accuracy of the model was verified. *SIRT3* was a promising biomarker for AMI diagnosis. Previous studies have demonstrated that *SIRT3* acts as a protective factor against ischemic heart injury, and might prevent myocardial injury by targeting mitochondrial dysfunction (56, 57). Thus, *SIRT3* is suggested as a diagnostic biomarker and therapeutic target for patients with an AMI.

Furthermore, the GSVA results showed that adipogenesis, Myc targets, oxidative phosphorylation, and UV response were the four hallmark pathways between the two risk groups, which was in line with the previous findings that myocardial infarction is accompanied by the regulation of adipogenesis and the regulation of mitochondrial function contributes to preventing myocardial fat deposition (58). In addition, oxidative stress caused by dramatically increased ROS levels could promote adipogenesis and affects mitochondrial function maintenance (59), contributing to AMI progression. Myc is an increasingly recognized biomarker in mitochondrial diseases. Studies have revealed that Myc could drive the differentiation of adipocytes in the heart and is increased following cardiac ischemia to induce adipogenesis (60). These findings imply that oxidative stress and adipogenesis have a role in the pathogenesis of AMI in the high-risk group. According to the GO functional enrichment analyses, the MRDEGs were enriched in MFs such as ubiquitin or ubiquitin-like protein ligase binding, which was consistent with the previous findings that mitophagy eliminates dysfunctional or redundant mitochondria via either ubiquitin-independent or ubiquitin-dependent pathways (61). In addition, gene set enrichment analysis was conducted and revealed that the pathways related to transcriptional regulation of immune cell activity and ischemic injury were differentially expressed between the patients with an AMI and the control individuals, suggesting the importance of modulating the immune system and targeting ischemia in AMI.

Mitophagy is increasingly recognized as an effective way to control the immune system by directly regulating mitochondrial antigen presentation and immune cell homeostasis (18). Studies have revealed that mitochondrial dysfunction in immune cells can enhance the inflammatory response and suppress repair following myocardial infarction (62). Thus, utilizing mitophagy might provide novel therapeutic opportunities for AMI. In our study, we found different immune cell infiltration patterns in the high- and low-risk groups of AMI samples. Furthermore, we revealed a significant correlation between the expression of the key genes and the immune cell infiltration levels in the high- and low-risk groups, which indicated that the key MRDEGs were associated with the immune process in AMI.

The interaction networks of the key genes were also investigated. MiRNAs, transcription factors, and RBPs are important regulators of mRNA expression (63–65). The interaction networks among the key MRDEGs themselves or with miRNAs, TFs, RBPs, and drug compounds were generated. For example, miR-497-5p has been revealed to contribute to heart failure following an MI (66). In this study, we observed that miR-497-5p was predicted to regulate both *AGPS* and *PYGB*, which were also downregulated in AMI and might serve as targets of miR-497-5p in AMI. These findings regarding the regulatory network of key genes might deepen our understanding of the molecular basis of mitophagy in AMI progression and therapy.

This study also had some limitations. First, we explored the key mitophagy-related biomarkers in AMI based on publicly available data, and their diagnostic value should be validated in other datasets and using further experiments in the future. Second, the potential mechanisms of the selected key genes in AMI require further investigation. Third, although we have demonstrated the difference in immune infiltration patterns between the two risk groups, the difference in therapeutic response in the two risk groups remains unclear. Future research is needed to elucidate the potential mechanisms of the key genes for AMI diagnosis and therapy.

## 5 Conclusions

In conclusion, we identified 12 key mitophagy-related biomarkers in AMI and constructed a risk model for AMI diagnosis that had good performance. The association between immune cell infiltration and different AMI risk groups was explored, and mitophagy-related biomarkers were related to the immune response in AMI patients, which might provide clues for understanding the roles of MRGs in AMI. Future research is also required to elucidate the molecular mechanisms of these key MRDEGs in AMI.

## Data Availability

Publicly available datasets were analyzed in this study. This data can be found here https://www.ncbi.nlm.nih.gov/geo/, accession numbers GSE24519; GSE34198.
