# Supplementary material for: Identification of mitophagy-related key genes and their correlation with immune cell infiltration in acute myocardial infarction via bioinformatics analysis
Source: Front Cardiovasc Med. 2025 Jan 13;11:1501608. doi: 10.3389/fcvm.2024.1501608 (PMC11770045; doi:10.3389/fcvm.2024.1501608)
Supplement: Supplementary file 1 [file Table1.docx]

**SUPPLEMENTARY TABLE 1** **AMI Dataset information list.**

|  | GSE24519 | GSE34198 |
| --- | --- | --- |
| Platform | GPL4133 | GPL13667 |
| Species | Homo sapiens | Homo sapiens |
| Tissue | Blood | Blood |
| Samples in AMI group | 38 | 49 |
| Samples in Control group | 0 | 48 |
| Reference | / | / |

AMI, Acute myocardial infarction.

**SUPPLEMENTARY TABLE 2 GO and KEGG enrichment analysis results of MRDEGs.**

| ONTOLOGY | ID | Description | GeneRatio | BgRatio | Padj | qvalue |
| --- | --- | --- | --- | --- | --- | --- |
| CC | GO:0005774 | vacuolar membrane | 2023/6/28 | 449/19594 | 0.005707 | 0.003365 |
| CC | GO:0030666 | endocytic vesicle membrane | 2023/4/28 | 194/19594 | 0.007684 | 0.004532 |
| CC | GO:0005741 | mitochondrial outer membrane | 2023/4/28 | 205/19594 | 0.007684 | 0.004532 |
| CC | GO:0005765 | lysosomal membrane | 2023/5/28 | 404/19594 | 0.007684 | 0.004532 |
| CC | GO:0098852 | lytic vacuole membrane | 2023/5/28 | 404/19594 | 0.007684 | 0.004532 |
| CC | GO:0031968 | organelle outer membrane | 2023/4/28 | 232/19594 | 0.007684 | 0.004532 |
| CC | GO:0019867 | outer membrane | 2023/4/28 | 234/19594 | 0.007684 | 0.004532 |
| CC | GO:0005742 | mitochondrial outer membrane translocase complex | 2023/2/28 | 21/19594 | 0.008243 | 0.004861 |
| CC | GO:0098799 | outer mitochondrial membrane protein complex | 2023/2/28 | 22/19594 | 0.008243 | 0.004861 |
| CC | GO:0034774 | secretory granule lumen | 2023/4/28 | 322/19594 | 0.014652 | 0.008641 |
| CC | GO:0060205 | cytoplasmic vesicle lumen | 2023/4/28 | 325/19594 | 0.014652 | 0.008641 |
| CC | GO:0031983 | vesicle lumen | 2023/4/28 | 327/19594 | 0.014652 | 0.008641 |
| CC | GO:0030139 | endocytic vesicle | 2023/4/28 | 342/19594 | 0.014652 | 0.008641 |
| CC | GO:0005766 | primary lysosome | 2023/3/28 | 155/19594 | 0.014652 | 0.008641 |
| CC | GO:0042582 | azurophil granule | 2023/3/28 | 155/19594 | 0.014652 | 0.008641 |
| CC | GO:0030118 | clathrin coat | 2023/2/28 | 39/19594 | 0.014652 | 0.008641 |
| CC | GO:0005782 | peroxisomal matrix | 2023/2/28 | 50/19594 | 0.021007 | 0.012388 |
| CC | GO:0031907 | microbody lumen | 2023/2/28 | 50/19594 | 0.021007 | 0.012388 |
| MF | GO:0031625 | ubiquitin protein ligase binding | 2023/5/28 | 298/18410 | 0.007495 | 0.005479 |
| MF | GO:0044389 | ubiquitin-like protein ligase binding | 2023/5/28 | 317/18410 | 0.007495 | 0.005479 |
| MF | GO:0031386 | protein tag | 2023/2/28 | 13/18410 | 0.008266 | 0.006042 |
| MF | GO:0030515 | snoRNA binding | 2023/2/28 | 32/18410 | 0.038722 | 0.028306 |
| KEGG | hsa04137 | Mitophagy - animal | 2023/5/20 | 72/8164 | 3.70E-05 | 3.21E-05 |

GO: Gene Ontology; BP: biological process; CC: cellular component; MF: molecular function; KEGG, Kyoto Encyclopedia of Genes and Genomes; MRDEGs: Mitophagy related differentially expressed genes.

**SUPPLEMENTARY TABLE 3 GSEA enrichment analysis AMI Dataset (AMI/Control).**

| ID | setSize | enrichmentScore | NES | pvalue | Padj | qvalue | |
| --- | --- | --- | --- | --- | --- | --- | --- |
| LEONARD_HYPOXIA | 24 | 0.638005 | 1.918456 | 0.001247 | 0.041038 | | 0.037292 |
| NAKAJIMA_EOSINOPHIL | 14 | -0.87874 | -2.26705 | 0.000383 | 0.019745 | | 0.017943 |
| SARRIO_EPITHELIAL_MESENCHYMAL_TRANSITION_UP | 82 | -0.53267 | -2.02214 | 0.000367 | 0.019745 | | 0.017943 |
| BLANCO_MELO_BRONCHIAL_EPITHELIAL_CELLS_INFLUENZA_A_DEL_NS1_INFECTION_DN | 81 | -0.46444 | -1.75954 | 0.000734 | 0.031477 | | 0.028604 |
| LI_WILMS_TUMOR_ANAPLASTIC_UP | 11 | -0.82087 | -1.96471 | 0.00157 | 0.045945 | | 0.041751 |
| GRAHAM_CML_DIVIDING_VS_NORMAL_QUIESCENT_UP | 115 | -0.58128 | -2.33357 | 0.000365 | 0.019745 | | 0.017943 |
| LEE_EARLY_T_LYMPHOCYTE_UP | 57 | -0.64794 | -2.28713 | 0.000376 | 0.019745 | | 0.017943 |
| ROSTY_CERVICAL_CANCER_PROLIFERATION_CLUSTER | 77 | -0.58829 | -2.20651 | 0.000366 | 0.019745 | | 0.017943 |
| WINNEPENNINCKX_MELANOMA_METASTASIS_UP | 79 | -0.57478 | -2.16771 | 0.000365 | 0.019745 | | 0.017943 |
| ZHAN_MULTIPLE_MYELOMA_PR_UP | 18 | -0.78144 | -2.14054 | 0.000384 | 0.019745 | | 0.017943 |
| BENPORATH_PROLIFERATION | 71 | -0.57129 | -2.11072 | 0.000369 | 0.019745 | | 0.017943 |
| WHITFIELD_CELL_CYCLE_LITERATURE | 25 | -0.70346 | -2.09547 | 0.000384 | 0.019745 | | 0.017943 |
| RHEIN_ALL_GLUCOCORTICOID_THERAPY_DN | 196 | -0.48106 | -2.06875 | 0.000353 | 0.019745 | | 0.017943 |
| BURTON_ADIPOGENESIS_3 | 59 | -0.57812 | -2.05281 | 0.000373 | 0.019745 | | 0.017943 |
| SENGUPTA_NASOPHARYNGEAL_CARCINOMA_UP | 164 | -0.47841 | -2.01835 | 0.000356 | 0.019745 | | 0.017943 |
| DANG_MYC_TARGETS_UP | 58 | -0.5673 | -2.00861 | 0.000374 | 0.019745 | | 0.017943 |
| KONG_E2F3_TARGETS | 50 | -0.58232 | -2.00547 | 0.000376 | 0.019745 | | 0.017943 |
| CHEOK_RESPONSE_TO_HD_MTX_UP | 10 | -0.85945 | -2.00283 | 0.000397 | 0.019745 | | 0.017943 |
| CHIANG_LIVER_CANCER_SUBCLASS_UNANNOTATED_DN | 104 | -0.49697 | -1.96245 | 0.000367 | 0.019745 | | 0.017943 |

AMI, Acute myocardial infarction; GSEA: Gene Set Enrichment Analysis.

**SUPPLEMENTARY TABLE 4 GSVA enrichment analysis AMI Dataset (High/Low).**

| Description | logFC | AveExpr | pvalue | Padj | B |
| --- | --- | --- | --- | --- | --- |
| HALLMARK_ADIPOGENESIS | -0.159959407 | 0.012112092 | 0.000413711 | 0.020685544 | -0.165965633 |
| HALLMARK_OXIDATIVE_PHOSPHORYLATION | -0.147415487 | 0.009459346 | 0.009622239 | 0.240555982 | -2.966905146 |
| HALLMARK_MYC_TARGETS_V2 | -0.158959998 | 0.019312577 | 0.015204059 | 0.253400975 | -3.361297126 |
| HALLMARK_UV_RESPONSE_UP | -0.090875298 | -0.01323713 | 0.044360453 | 0.554505666 | -4.261488695 |

GSVA, Gene Set Variation Analysis; AMI, Acute myocardial infarction
